# Selective Autophagy Maintains the Aryl Hydrocarbon Receptor Levels in HeLa Cells: A Mechanism That Is Dependent on the p23 Co-Chaperone

**DOI:** 10.3390/ijms21103449

**Published:** 2020-05-13

**Authors:** Yujie Yang, William K. Chan

**Affiliations:** Department of Pharmaceutics & Medicinal Chemistry, Thomas J. Long School of Pharmacy, University of the Pacific, Stockton, CA 95211, USA; y_yang16@u.pacific.edu

**Keywords:** AHR, macroautophagy, p23, LC3, p62

## Abstract

The aryl hydrocarbon receptor (AHR) is an environmental sensing molecule which impacts diverse cellular functions such as immune responses, cell growth, respiratory function, and hematopoietic stem cell differentiation. It is widely accepted that the degradation of AHR by 26S proteasome occurs after ligand activation. Recently, we discovered that HeLa cells can modulate the AHR levels via protein degradation without exogenous treatment of a ligand, and this degradation is particularly apparent when the p23 content is down-regulated. Inhibition of autophagy by a chemical agent (such as chloroquine, bafilomycin A1, or 3-methyladenine) increases the AHR protein levels in HeLa cells whereas activation of autophagy by short-term nutrition deprivation reduces its levels. Treatment of chloroquine retards the degradation of AHR and triggers physical interaction between AHR and LC3B. Knockdown of LC3B suppresses the chloroquine-mediated increase of AHR. Down-regulation of p23 promotes AHR degradation via autophagy with no change of the autophagy-related gene expression. Although most data in this study were derived from HeLa cells, human lung (A549), liver (Hep3B), and breast (T-47D and MDA-MB-468) cells also exhibit AHR levels sensitive to chloroquine treatment and AHR–p62/LC3 interactions. Here we provide evidence supporting that AHR undergoes the p62/LC3-mediated selective autophagy in HeLa cells.

## 1. Introduction

The aryl hydrocarbon receptor (AHR) is originally recognized as a transcription factor in response to environmental toxicants such as dioxins and polycyclic aromatic hydrocarbons [1]. Since then, more roles of AHR such as its involvement in organ development [2], immune responses [3,4], nervous system homeostasis [5], and carcinogenesis [6,7] have been reported. Not surprisingly, AHR has become a promising target for the treatment of diseases such as autoimmune diseases, inflammation, and cancers. However, the AHR function is often ligand-, cell context-, and tissue-specific, confounding the feasibility of targeting AHR for rational drug design. Understanding the basic biology of AHR would provide much-needed information for effective modulation of the AHR function.

AHR resides in the cytoplasm as a complex with HSP90, XAP2, and p23. The p23 co-chaperone is in a variety of HSP90 client protein complexes [8,9,10,11]. It has been reported that p23 stabilizes the ATP-induced conformation of the HSP90-AHR complex [12,13]. The AHR function can also be enhanced by p23 in vitro [12,14,15,16], but surprisingly this enhancement is not observed in the p23-null mouse embryo [17], possibly due to yet unidentified compensatory mechanisms. The effect of p23 on AHR is largely uncharacterized at present.

Autophagy is essential in maintaining cell homeostasis by delivering cytosolic cargos to lysosomes for degradation. There are three types of autophagy: macroautophagy which involves the formation of autophagosome, followed by its fusion with lysosome; microautophagy which involves substrates that are directly sequestered via lysosomal invagination; and lastly, chaperone-mediated autophagy which involves the HSC70-mediated delivery of client proteins into lysosome via interaction with LAMP-2A (for general review, see [18]). Although macroautophagy is generally known to deliver bulk cargos to lysosomes in a non-selective manner, it can also be selective in delivering cargo proteins for lysosomal degradation. Recognition of client proteins by cargo receptors (such as p62) provides the selectivity of macroautophagy. This recognition, in most cases, is mediated through the ubiquitination of client proteins. Different linkages of ubiquitin destine the ubiquitinated proteins to different degradation pathways; for example, K63 ubiquitination is usually recognized by cargo receptors to undergo lysosomal degradation whereas proteins with K48 ubiquitination are specifically delivered to 26S proteasome for degradation [19]. After K63 ubiquitination of a client protein, LC3B-II, a phosphatidylethanolamine-conjugated, membrane-bound form of cleaved LC3B-I [20], forms the autophagosome with the client protein–cargo receptor complex, leading to lysosomal degradation of the client protein [20,21].

Upon binding of a ligand, AHR translocates into the nucleus and activates gene transcription. The liganded AHR is subsequently degraded by the ubiquitin–proteasome system to limit its transactivation function [22]. However, how AHR is regulated in the absence of exogenous ligand treatment remained unclear. We previously discovered that down-regulation of just half of the p23 cellular content is sufficient in causing a lower cellular content of AHR in various cancer and untransformed cell types in vitro [15,16,23]. AHR is degraded significantly faster in the p23 knockdown Hepa1c1c7 cells; however, inhibition of proteasome activity by MG132 cannot restore the AHR protein levels, suggesting that AHR is not degraded by the 26S proteasome when p23 is down-regulated [15]. Treatment of MG132, surprisingly, decreases the AHR protein levels in both wild type and p23 knockdown Hepa1c1c7 cells, suggesting that mechanisms in degrading the unliganded AHR protein could be enhanced by proteasomal inhibition. It has been reported that crosstalk occurs between the ubiquitin–proteasome system (UPS) and autophagy [24,25]. Blockade of UPS leads to protein aggregation, which in turn triggers the autophagy-mediated protein degradation [26]. In addition, reduced amino acids in cells as a consequence of MG132 treatment can activate autophagy [27]. Thus, we explored whether the autophagy-lysosomal system is responsible for the degradation of AHR without exogenous ligand treatment. Here we provide evidence to support that the basal AHR protein levels in HeLa cells are controlled by selective autophagy. Knockdown of p23 reduces AHR protein levels by promoting the K63-linked ubiquitination of AHR, followed by the p62/LC3-mediated AHR degradation.

## 2. Results

### 2.1. Autophagy Activity in Wild Type and p23 Stable Knockdown HeLa Cells Governs the Levels of AHR

**Figure 1:**
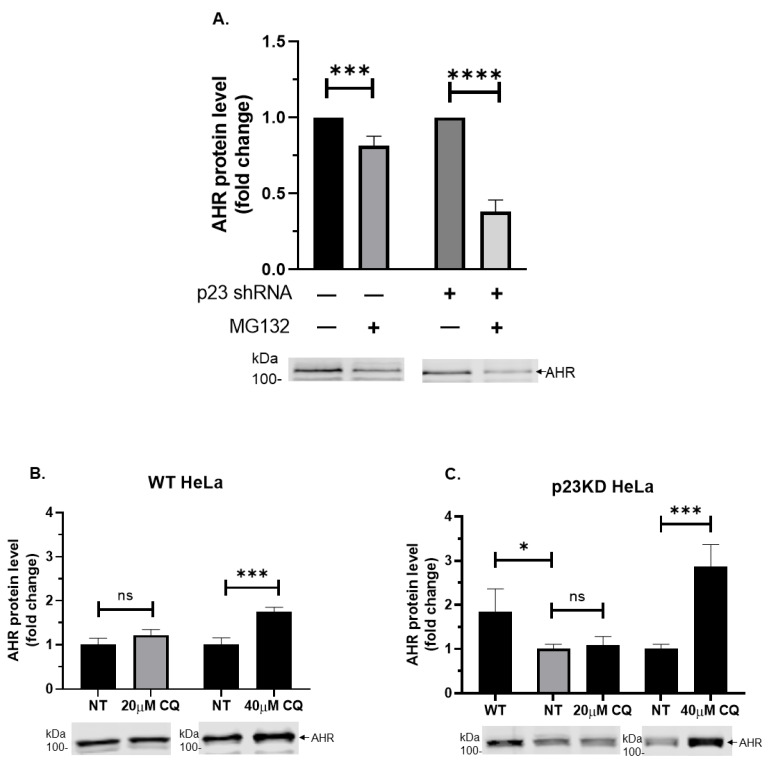

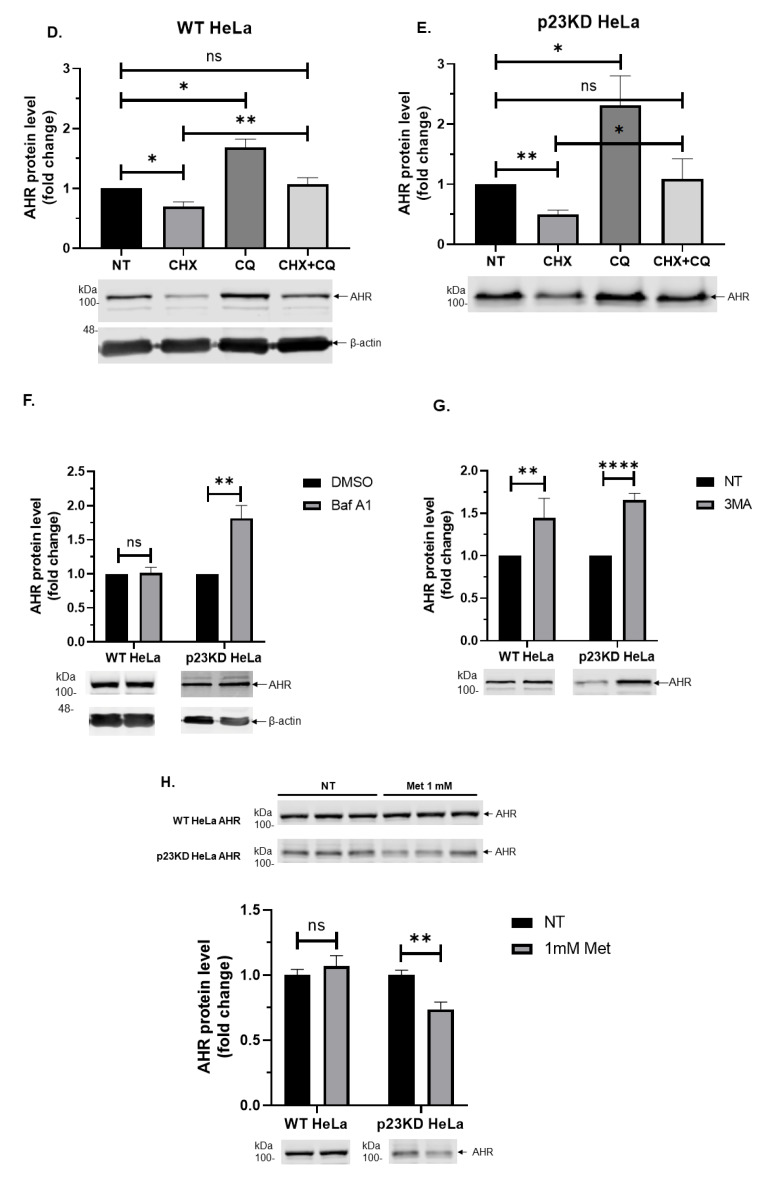
Using an autophagy inhibitor or activator to reveal that the aryl hydrocarbon receptor (AHR) undergoes autophagy in wild type (WT) and p23 stable knockdown (p23KD) HeLa cells. (**A**) MG132 (10 μM for 6 h) decreased AHR protein levels more in HeLa cells transiently transfected with p23 shRNA (2.5 μg for 48 h) than in WT HeLa cells. EndoFectin transfection reagent (2:1) was used. The below images are representative of the replicate data (means ± SD, *n* = 4). Conditions with no MG132 treatment were arbitrarily set as one (with no error bar) for data normalization. Multiple *t*-tests corrected with the Holm-Sidak method for multiple comparisons were performed to determine statistical significance. (**B**) WT and (**C**) p23KD HeLa cells were treated with 20 μM or 40 μM chloroquine (CQ) dissolved in water for 6 h. Western analysis results showed a higher increase of AHR in p23KD than in WT HeLa cells after 40 µM CQ treatment. For (**B**,**C**), the below images are representative of the replicate data of one experiment (means ± SD, *n* = 3). Conditions with no addition as no treatment (NT) were arbitrarily set as one for comparison. This experiment was repeated once with similar results. One-way ANOVA with Sidak’s multiple comparisons test was performed to determine statistical significance. (**D**) WT and (**E**) p23KD HeLa cells were treated with 40 μg/mL of cycloheximide (CHX) for 6 h in the presence or absence of 40 μM CQ for 12 h (6 h pre-treatment and then co-treated with CHX for another 6 h). The degradation of AHR in both cell lines was inhibited by CQ. For (**D**,**E**), the below images are representative of the replicate data (means ± SD, *n* = 3). Conditions with no addition as no treatment (NT) were arbitrarily set as one (with no error bar) for data normalization. Multiple *t*-tests corrected with the Holm-Sidak method for multiple comparisons were performed to determine statistical significance. (**F**) Treatment of 6 nM Bafilomycin A1 (Baf A1) dissolved in DMSO for 3 h increased AHR protein levels only in p23KD cells but not WT HeLa cells. (**G**) Treatment of 5 mM 3-methyladenine (3MA) dissolved in DMEM for 24 h increased AHR protein levels in WT and p23KD HeLa cells. For (**F**,**G**), the below images are representative of the replicate data (means ± SD, *n* = 3 for (**F**), *n* = 4 for (**G**)). Conditions with DMSO treatment and no addition as no treatment (NT) of WT and p23KD were arbitrarily set as one (with no error bar) for data normalization. Multiple *t*-tests corrected with the Holm-Sidak method for multiple comparisons were performed to determine statistical significance. (**H**) Treatment of 1 mM metformin (Met) for 4 h decreased AHR protein levels in p23KD HeLa cells but not in WT HeLa cells. The above images represent the replicate data of one experiment (means ± SD, *n* = 3). Conditions with no treatment (NT) were arbitrarily set as one for comparison. This experiment was repeated once with similar results. Multiple *t*-tests corrected with the Holm-Sidak method for multiple comparisons were performed to determine statistical significance. For (**A**–**H**), each Western lane contained 30 μg of whole-cell lysate. Data in (**D**,**F**) were normalized by β-actin (as shown) whereas the rest of the data were normalized by total protein stain.

We previously observed that suppression of the AHR levels after down-regulation of p23 in Hepa1c1c7 mouse hepatoma cells was caused by increased AHR degradation, which could not be reversed by the treatment of a proteasome inhibitor MG132 [15]. In this study, we observed that MG132 similarly reduced the AHR content in wild type and p23 knockdown (human cervical) HeLa cells in a statistically significant manner (Figure 1A). We then explored whether this AHR degradation could be mediated through autophagy-mediated lysosomal degradation. We treated the HeLa cells with an autophagy inhibitor CQ at 20 or 40 μM for six hours. We observed that the AHR protein levels in both wild type and p23 stable knockdown HeLa cells were increased in the presence of 40 µM CQ (Figure 1B,C). The cellular AHR protein levels in p23 stable knockdown HeLa cells was about 54% when compared to that of wild type HeLa cells (Figure 1C, “WT” versus “NT”). However, the AHR levels in p23 stable knockdown cells became even higher than the levels in wild type cells after treatment of 40 μM CQ for six hours, suggesting that mechanism of the p23-mediated AHR degradation can be reversed by CQ. Next, we performed the cycloheximide experiment to examine whether degradation of AHR is mediated through autophagy. Basically, when protein synthesis in HeLa cells is blocked by cycloheximide, any reduction of the AHR content is the consequence of protein degradation. If this degradation is mediated through autophagy, CQ should, in principle, reverse the AHR reduction in the presence of cycloheximide. Indeed, we observed that degradation of the AHR protein in p23 stable knockdown and wild type HeLa cells was inhibited in the presence of CQ (Figure 1D,E, “CHX” versus “CHX+CQ”). Two other autophagy inhibitors, namely Baf A1 and 3MA, also increased the AHR protein levels in p23 stable knockdown HeLa cells (Figure 1F,G). However, only 5 mM 3MA, but not 6 nM Baf A1, increased the AHR levels in wild type HeLa cells. An autophagy inducer metformin decreased the AHR protein levels in p23 knockdown but not in wild type HeLa cells at 1 mM concentration (Figure 1H). We cannot rule out the possibility that a higher concentration of Baf A1 and metformin is necessary for eliciting changes of the AHR levels in wild type HeLa cells. Nonetheless, these data supported that the basal AHR undergoes the autophagy-mediated degradation in HeLa cells and this degradation is more apparent when p23 is down-regulated.

### 2.2. Short-Term Nutrient Deprivation Triggers Degradation of AHR in HeLa Cells When Either p23 or HSP90 is Down-Regulated

**Figure 2:**
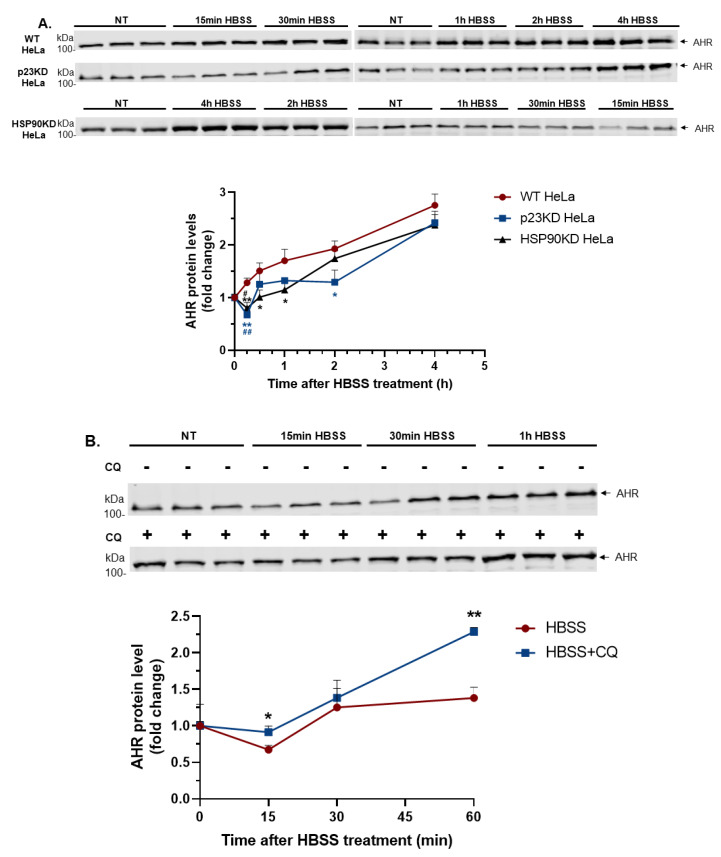

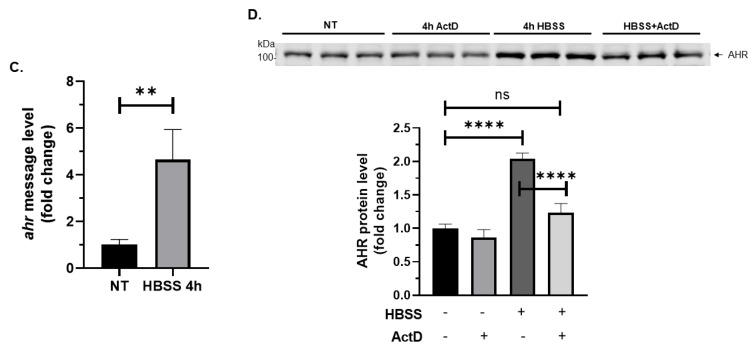
Short-term nutrient deprivation triggers the degradation of AHR in HeLa cells when either p23 or HSP90 is down-regulated. (**A**) Zero to four hours’ treatment of HBSS (nutrient deprivation) in wild type (WT), p23 stable knockdown (p23KD), and HSP90 stable knockdown (HSP90KD) HeLa cells. Fifteen minutes of HBSS treatment decreased AHR protein levels in p23KD and HSP90 HeLa cells but not in WT HeLa cells. Longer nutrient deprivation of up to 4 h increased AHR protein levels in all three cell lines. The graph represents replicate data of means ± SD (upper error bars shown), *n* = 3 for all, except *n* = 4 for HSP90KD data from 0 to 1 h. Zero timepoints in each cell line were arbitrarily set as one for comparison. Data were analyzed by unpaired two-tailed *t*-test for comparisons between zero and 15 min timepoints in each individual cell line (significant data points marked with # symbol). Data were analyzed by two-way ANOVA (mixed-model) analysis corrected with the Dunnett test for multiple comparisons to determine the statistical significance between WT and p23KD or WT and HSP90 KD at each time point (significant data points marked with * symbol). WT HeLa data were set as a control group at each timepoint for comparison. The images above represent the replicate data. Note that the timepoints are intentionally presented not in chronological order to preserve the integrity of the original immunoblots. (**B**) A 40 μM amount of CQ pre-treatment for 6 h blocked the AHR decrease in p23KD HeLa cells treated with HBSS for 0–1 h. Zero time points in each condition were arbitrarily set as one for comparison. The graph represents replicate data of means ± SD (upper error bars shown), *n* = 3. Data were analyzed by multiple *t*-tests corrected with the Holm-Sidak method for multiple comparisons to determine the statistical significance. The images above represent the replicate data. (**C**) A 4 h HBSS treatment significantly increased the *ahr* message levels in WT HeLa cells. The graph represents replicate data of means ± SD, *n* = 3 of one experiment. This experiment was repeated once with similar results. Data were analyzed by unpaired *t*-test to determine statistical significance. (**D**) Treatment of 5 μg/mL of actinomycin D (ActD) for 4 h abolished the increase of AHR protein levels induced by HBSS (nutrient deprivation) in WT HeLa cells. The graph represents replicate data of means ± SD, *n* = 3 of one experiment. This experiment was repeated once with similar results. Data were analyzed by one-way ANOVA with Tukey’s multiple comparisons test to determine statistical significance. For A to D, each Western lane contained 30 μg of whole-cell lysate. The intensity of all Western bands was normalized by total protein stain.

To further investigate whether autophagy is involved in the degradation of AHR, we treated the HeLa cells with HBSS to mimic nutrient deprivation, which is known to cause autophagy. We observed that the AHR protein levels were reduced after 15 min of HBSS treatment and were then gradually increased up to four hours in p23 knockdown HeLa cells (Figure 2A). In contrast, the AHR protein levels of wild type HeLa cells were steadily increased up to four hours of HBSS treatment. Pre-treatment of CQ hampered the reduction of AHR in p23 knockdown HeLa cells, suggesting that this decrease of AHR protein levels is caused by autophagy (Figure 2B). We hypothesized that some compensatory mechanism may be involved in protecting AHR from degradation under nutrition deprivation in HeLa cells. Given that HSP90 stabilizes the cytosolic AHR complex, we examined whether HSP90 would protect AHR from degradation caused by nutrient deprivation in HeLa cells. We down-regulated HSP90 in HeLa cells with shRNA via lentiviral infection and then treated these HSP90 knockdown cells with HBSS. We observed that these cells exhibited similar characteristics as the p23 knockdown cells but not as the wild type cells, suggesting that this decrease of AHR could be protected by HSP90 (Figure 2A). qPCR data showed that 4 h HBSS treatment significantly increased the amount of the *ahr* message in wild type HeLa cells (Figure 2C). Co-treatment of a transcription inhibitor actinomycin D with HBSS in wild type HeLa cells abolished the increase of AHR protein levels caused by HBSS (Figure 2D). Collectively, these data supported that although nutrient deprivation caused degradation of AHR via autophagy initially, it increased the synthesis of the AHR protein, which led to the steady rise of the AHR protein levels up to four hours of nutrient deprivation.

### 2.3. Down-Regulation of p23 in HeLa Cells Exhibits Higher Autophagic Flux

**Figure 3:**
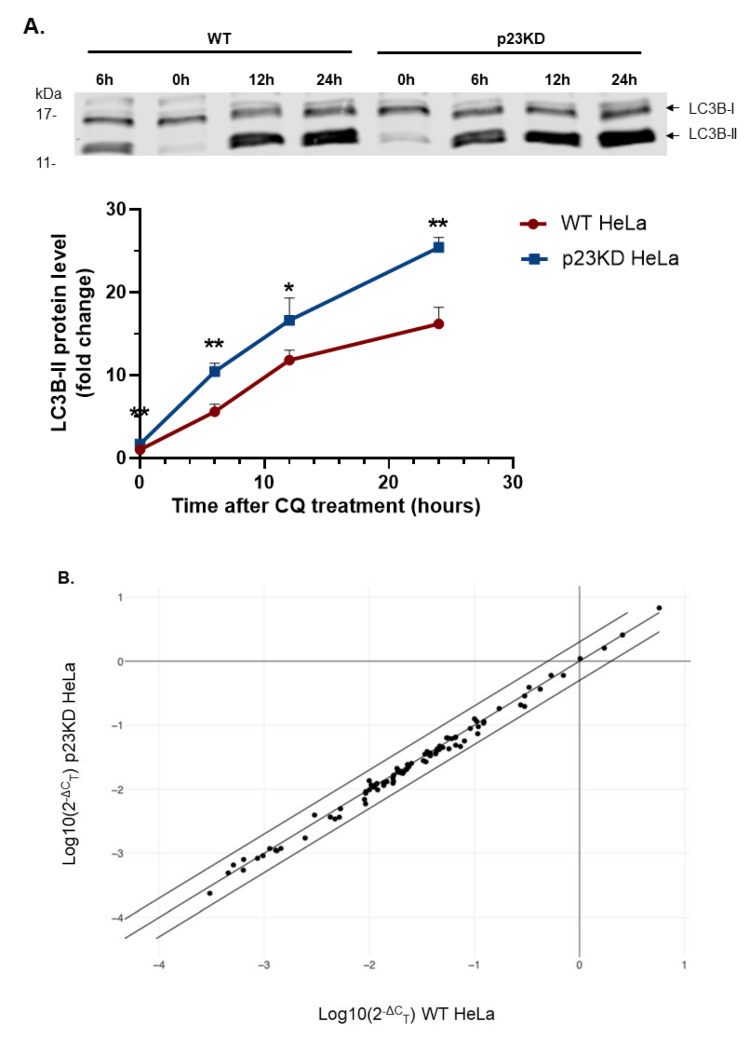
Down-regulation of p23 in HeLa cells exhibits higher autophagic flux. (**A**) Western blot analysis showing that p23 stable knockdown (p23KD) HeLa cells have higher basal LC3B-II protein levels as well as higher autophagic flux than wild type (WT) HeLa cells in the presence of 40 μM CQ. The graph in A represents replicate data of means ± SD (upper error bars shown), *n* = 3 of one experiment. This experiment was repeated once with similar results. Data were analyzed by multiple *t*-tests corrected with the Holm-Sidak method for multiple comparisons to determine the statistical significance. The above images are representative of the replicate data. Note that the hour under WT is intentionally presented as “6 h, 0 h, 12 h, 24 h” to preserve the integrity of the original immunoblot. Each Western lane contained 30 μg of whole-cell lysate. The intensity of Western bands was normalized by the total protein stain. (**B**) Qiagen RT^2^ Profiler PCR array analysis of 84 autophagy-related gene expression showing that knockdown of p23 did not alter the autophagy-related gene transcription when compared to wild type (WT). The scatter plot compares WT HeLa (*x*-axis) to p23 knockdown (p23KD) HeLa (*y*-axis) with a fold regulation setting as 2.

Next, we examined whether knockdown of p23 in HeLa cells stimulates autophagy, which in turn accelerates the degradation of the AHR protein. It is well accepted that LC3B-II plays a key role in macroautophagy [28]. Its turnover can be used as a marker for autophagic flux, which reflects the frequency of events from autophagosome formation to substrate degradation during macroautophagy. In other words, the amount of LC3B-II in the autophagosomes and lysosomes is directly proportional to the autophagy activity, which is referred to as the autophagic flux. We measured the LC3B-II protein levels and observed that the LC3B-II band was more intense in p23 knockdown HeLa cells than in wild type cells (Figure 3A, “WT, 0 h” versus “p23KD, 0 h”). Since CQ inhibits the LC3B-II degradation by acidic proteases in the lysosomes, the LC3B-II content can be more easily detected in the presence of CQ. The autophagic flux, which was determined by the slope of LCB-II levels over time with CQ treatment, was also higher in p23 knockdown than in wild type cells (Figure 3A), suggesting that down-regulation of p23 in HeLa cells sensitized macroautophagy. Next, we performed a PCR array to test whether knockdown of p23 would change the expression of autophagy-related genes. The expression of 84 autophagy-related genes (Table S1) was measured using the Qiagen RT^2^ Profiler PCR array kit. However, the gene expression related to autophagy was not significantly different between wild type and p23 knockdown HeLa cells (Figure 3B), suggesting that the down-regulation of p23 leads to higher autophagic activity without affecting the autophagy-related gene expression.

### 2.4. AHR Undergoes Selective Autophagy in p23 Knockdown and Wild Type HeLa Cells

**Figure 4:**
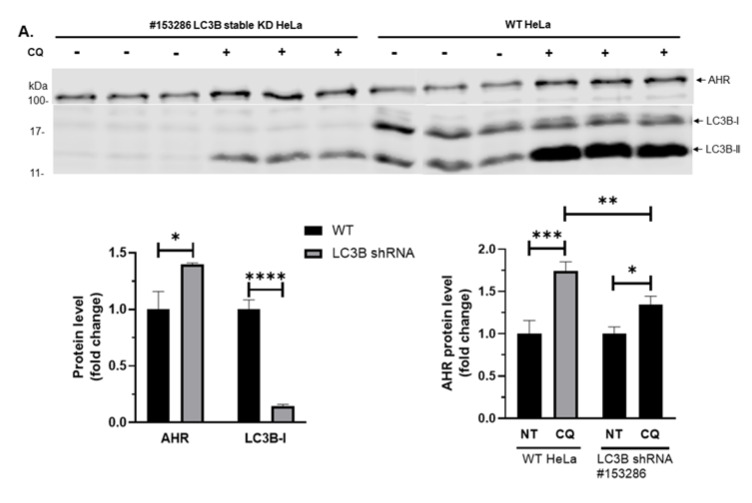

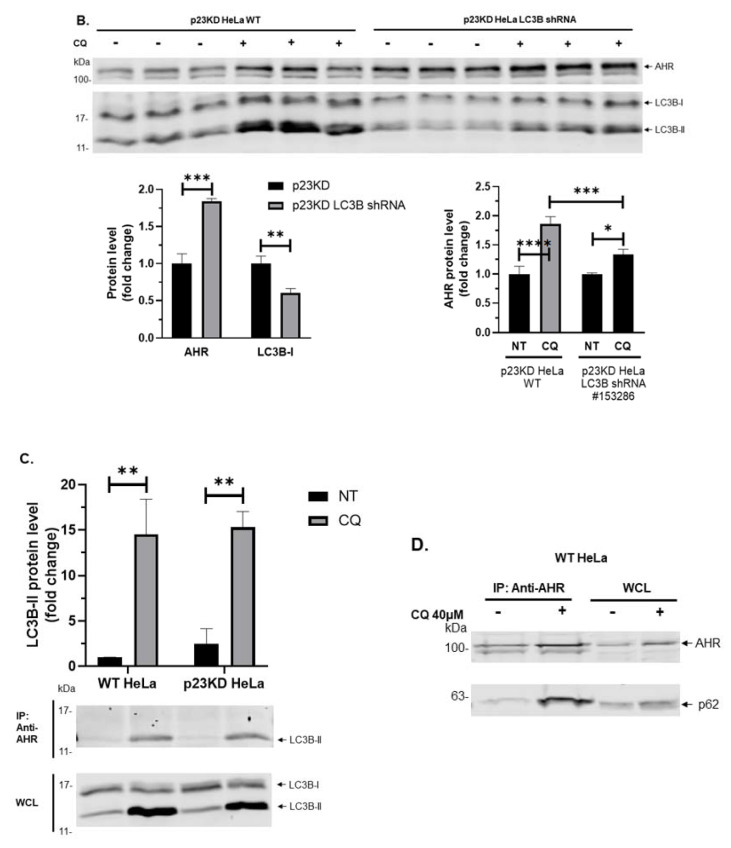
AHR interacts with LC3B-II and p62 in p23 stable knockdown (p23KD) and wild type (WT) HeLa cells. (**A**) Stable knockdown of LC3B in HeLa cells increased AHR protein levels and reduced the extent of the CQ-mediated increase of AHR. The images above show LC3B-I, LC3B-II, and AHR protein levels in WT and LC3B stable knockdown (KD) HeLa cells with or without treatment of 40 μM CQ for 6 h of triplicate samples. The graphs represent replicate data of means ± SD of one experiment, *n* = 3. WT (left graph) and NT (right graph) were arbitrarily set as 1 for data normalization. This experiment was repeated once with similar results. Data of the left graph were analyzed by multiple *t*-tests corrected with the Holm-Sidak method for multiple comparisons to determine statistical significance whereas data of the right graph were analyzed by one-way ANOVA with Sidak’s multiple comparisons test to determine statistical significance. (**B**) Transient knockdown of LC3B in p23 stable knockdown (p23KD) HeLa cells showed a higher increase of AHR protein levels when compared to that in WT HeLa cells (see A) and reduced the extent of CQ-mediated increase of AHR. The graphs represent replicate data of means ± SD of one experiment, *n* = 3. p23KD (left graph), and no addition as no treatment (NT, right graph) were arbitrarily set as 1 for data normalization. This experiment was repeated once with similar results. Data of the left graph were analyzed by multiple *t*-tests corrected with the Holm-Sidak method for multiple comparisons to determine statistical significance whereas data of the right graph were analyzed by one-way ANOVA with Sidak’s multiple comparisons test to determine statistical significance. (**C**) LC3B-II was co-immunoprecipitated by AHR polyclonal antibody SA210 in both cell lines after treatment of 40 μM CQ for 6 h. Data were presented from three independent experiments as means ± SD, *n* = 3. WT HeLa NT group was arbitrarily set as one for comparison (no error bar). Data were analyzed by multiple *t*-tests corrected with the Holm-Sidak method for multiple comparisons to determine statistical significance. The images below are representative of the replicate data. (**D**) p62 was co-immunoprecipitated by AHR monoclonal antibody A-3x after treatment of 40 μM CQ for 6 h in WT HeLa cells. This experiment was repeated once with similar results. For A-B, each Western lane contained 30 μg of whole-cell lysate or the whole immunoprecipitation content from 1 to 2 mg of whole-cell lysate starting material (C and D). Data were normalized by total protein stain.

Thus far, our data suggested possibly an LC3B-II-dependent lysosomal degradation of AHR in HeLa cells—this type of degradation has been described as selective (macro)autophagy. The selectivity of autophagy is mainly conveyed by the specific binding of cargo proteins to cargo receptors (e.g., p62) and the binding of LC3B-II to the cargo protein–cargo receptor complex [29]. We first examined the role of LC3B in the degradation of AHR in HeLa cells. Stable knockdown of LC3B gene (*MAP1LC3B*) in HeLa cells to 12.5% of the wild type LCB-I content showed an increase of the AHR protein levels when compared to the wild type HeLa cells (Figure 4A, left graph). In addition, knockdown of LC3B led to less LC3B-II formed from LC3B-I lipidation and suppressed the CQ effect on AHR, consistent with our suspicion that CQ might block the LC3B-mediated degradation of the AHR protein (Figure 4A,B, right graphs). Next, we transiently down-regulated 40% of the LC3B-I content in p23 knockdown HeLa cells and observed a higher increase of AHR when compared to the LC3B knockdown HeLa cells (Figure 4A,B, left graphs). The response of AHR to CQ treatment was also significantly suppressed by the LC3B knockdown (Figure 4B, right graph). Next, we examined whether AHR physically interacts with LC3B-II, leading to selective autophagy of the AHR protein. We observed that LC3B-II interacted with AHR in our coimmunoprecipitation experiment and this interaction can be significantly enhanced by CQ in both wild type and p23 knockdown HeLa cells (Figure 4C). There was also a significant amount of p62 co-immunoprecipitated with AHR (Figure 4D).

**Figure 5:**
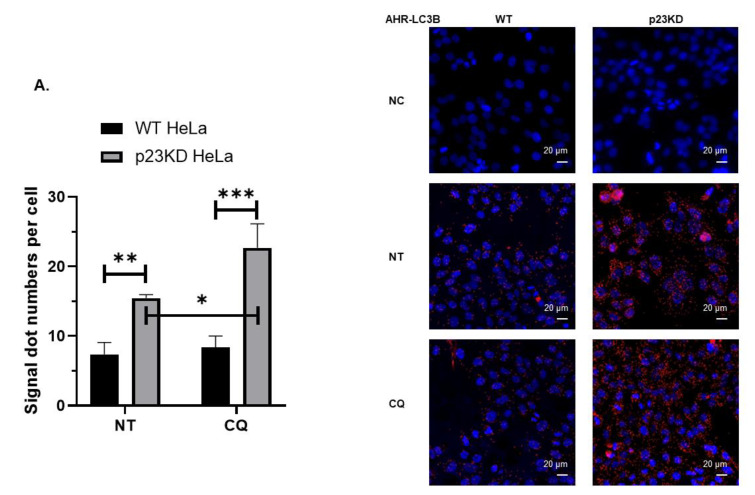

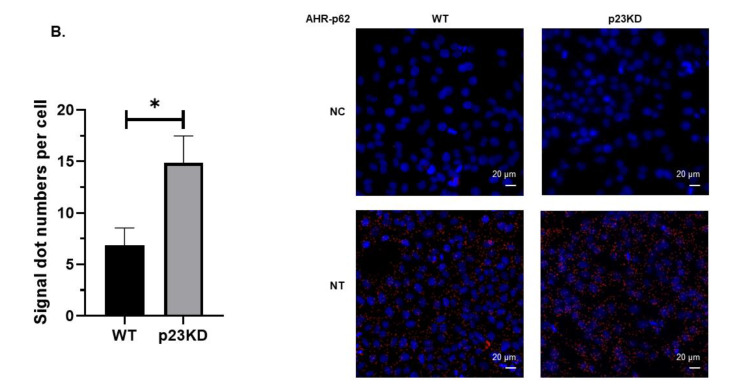
Proximity ligation assay results showing that AHR interacts with LC3B and p62 in wild type (WT) and p23 stable knockdown (p23KD) HeLa cells. (**A**) More AHR–LC3B interaction was detected in p23KD than in WT HeLa cells (NT images) and treatment of 40 μM CQ for 6 h increased the interaction (CQ versus NT images). (**B**) p23KD cells showed more AHR–p62 interaction than in WT HeLa cells (NT images). The graphs represent replicate data of means ± SD of one experiment, n=3. This experiment was repeated twice with similar results. Image J software was used to measure signal dots (red channel) and cell numbers (nucleus numbers in the blue channel) in each of the whole images captured (signals and nucleus on the edges were excluded). Statistical significance was determined by two-way ANOVA with Tukey’s multiple comparisons test (A) and unpaired *t*-test (B). The images are representative of the replicate data with a scale bar at the right bottom of each image. NC, negative control (cells incubated without antibodies against interaction partners); NT, no addition as no treatment; CQ, chloroquine in water.

**Figure 6:**
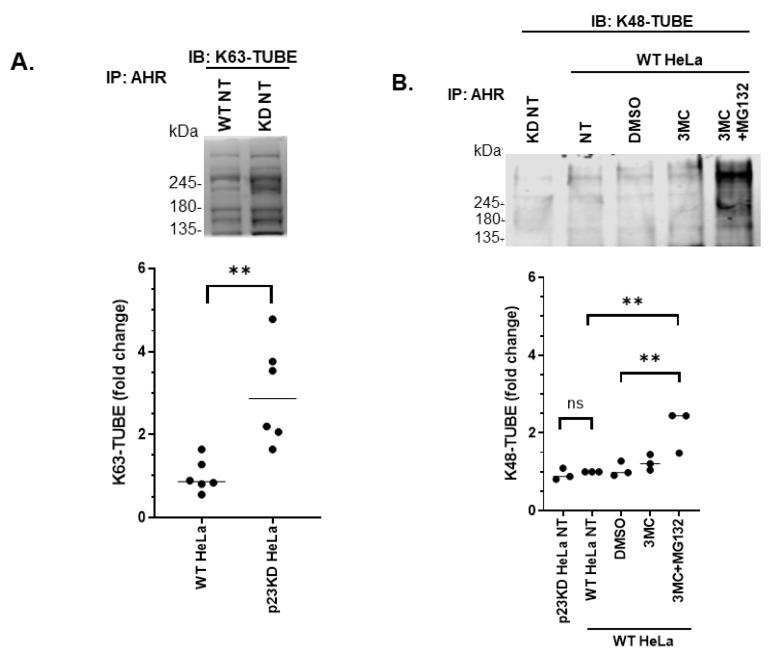
AHR undergoes K63 ubiquitination in p23 stable knockdown (p23KD) and wild type (WT) HeLa cells. (**A**) Immunoprecipitation of AHR by AHR polyclonal antibody SA210, followed by K63-TUBE Far-western analysis showing more K63-ubiquitinated AHR in p23KD than in WT HeLa cells. The graph represents replicate data (means ± SD, *n* = 6). The mean of WT HeLa was arbitrarily set as one for comparison. Data were analyzed by unpaired *t*-test to determine statistical significance. The images above are representative of the replicate data. (**B**) K48-TUBE was used for Far-western analysis showing minimal K48-ubiquitination of AHR in WT and p23KD HeLa cells. Treatment of 3MC (1 μM) and MG132 (10 μM) for 2 h showed accumulation of K48-ubiquitinated AHR. The pattern of K48-ubiquitinated AHR was different from K63-ubiquitinated AHR. The graph represents replicate data (means ± SD, *n* = 3). One WT HeLa NT value was arbitrarily set as one for comparison. Data were analyzed by one-way ANOVA with Tukey’s multiple comparisons test to determine statistical significance. The images above are representative of the replicate data. “WT NT” stands for wild-type HeLa cells with no treatment whereas “KD NT” stands for p23 knockdown HeLa cells with no treatment.

Proximity ligation assay was performed to confirm whether AHR would physically interact with LC3B and p62 in HeLa cells in situ. We observed that AHR interacted with LC3B (Figure 5A) and p62 (Figure 5B) in both wild type and p23 knockdown HeLa cells, whereas negative control groups without the addition of antibodies against the interaction partners did not show signals. There were more AHR–LC3B and AHR–p62 interactions in p23 knockdown than in wild type HeLa cells (Figure 5A,B). We expected that the signal of the LC3B-AHR interaction should be enhanced in the presence of CQ since LC3B-II can be accumulated when acidic proteases in the lysosomes are inhibited by CQ. Indeed, treatment of CQ enhanced the AHR–LC3B interaction significantly in p23 knockdown HeLa cells but not in WT HeLa cells (Figure 5A). Macroautophagy (or autophagy) can selectively degrade K63-ubiquitinated target proteins [30]. Given that we observed an interaction between AHR and p62, which is a known ubiquitin-binding cargo receptor, we examined the ubiquitination status of AHR in wild type and p23 knockdown HeLa cells. We observed that AHR was K63-ubiquitinated in both cell lines without exogenous ligand treatment and significantly more K63-ubiquitination was detected in p23 knockdown HeLa cells (Figure 6A). Both cell lines showed minimal levels of K48-ubiquitinated AHR protein levels (Figure 6B, “KD NT” and “NT” lanes of image). Co-treatment of an AHR ligand 3MC in the presence of a proteasome inhibitor MG132 showed a more intense K48-ubiquitinated AHR protein (Figure 6B), which exhibited a pattern that was different from the K63-ubiquitination of AHR as shown in Figure 6A. Collectively, these data supported that the basal AHR protein undergoes K63-ubiquitination (but not K48-ubiquitination) in HeLa cells and this K63-ubiquitination is more apparent when p23 is down-regulated. AHR undergoes p62/LC3-mediated selective autophagy via K63-ubiquitination in the absence of ligand treatment. Down-regulation of p23 promotes this degradation process, leading to a decrease in the basal AHR protein levels.

### 2.5. AHR is Degraded Via Autophagy in Human Lung, Liver, and Breast Cancer Cell Lines

**Figure 7:**
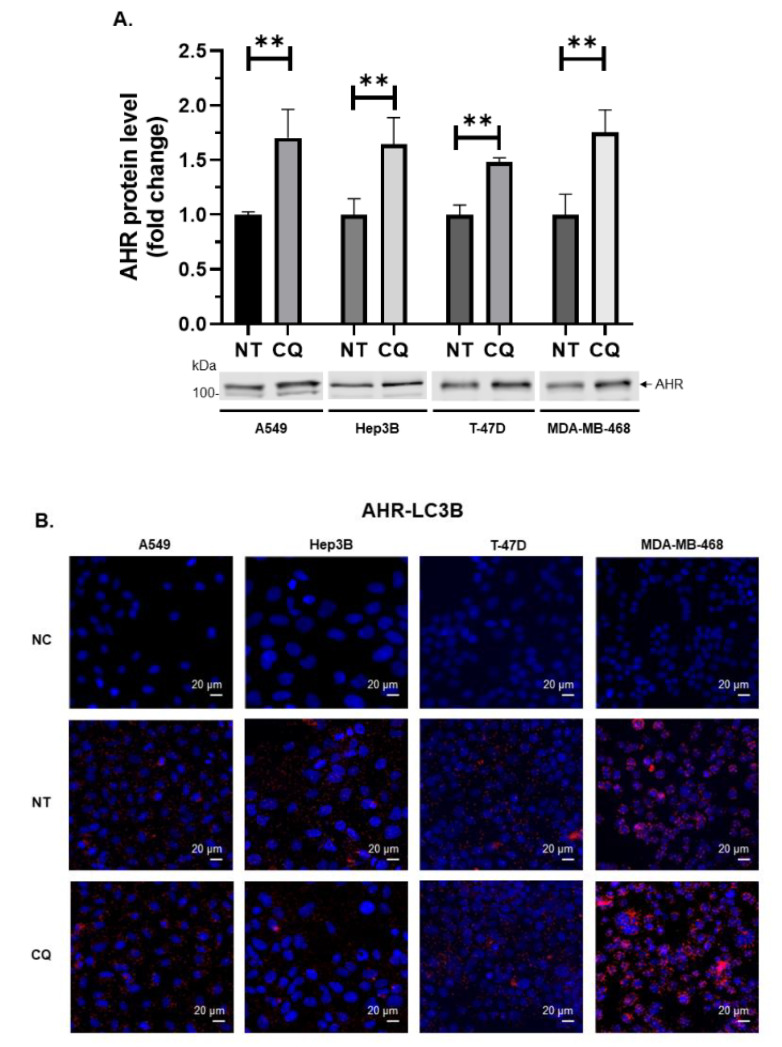

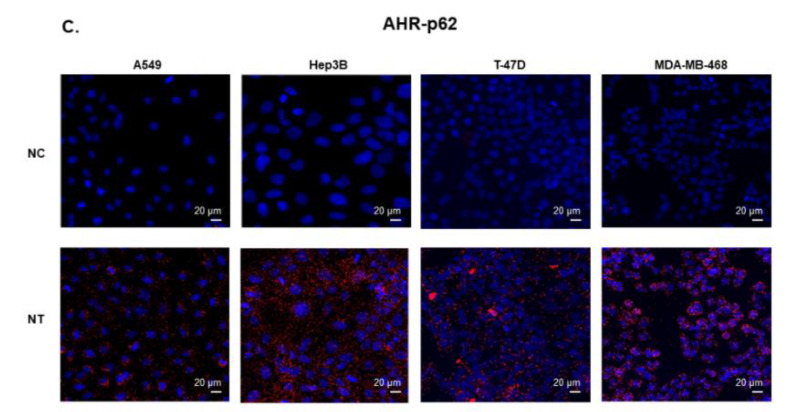
AHR is degraded via autophagy in human lung, liver, and breast cancer cell lines. (**A**) Treatment of 40 μM CQ dissolved in water for 6 h increased AHR protein levels in A549, Hep3B, T-47D, and MDA-MB-468 cells (means ± SD; *n* = 3 of one experiment, except for *n* = 5 for Hep3B). This experiment was repeated once with similar results. Each Western lane contained 30 μg of whole-cell lysate. Data are normalized by the total protein stain. NT, no addition as no treatment. An unpaired *t*-test was used to determine statistical significance. The images below are representative of the replicate data. Proximity ligation assay results showing that AHR interacted with (**B**) LC3B and (**C**) p62 in all four cell lines. All images are representative of the replicate data of three independent experiments. Each image contains a scale bar at the right bottom. NC, negative control (cells incubated without primary antibodies against interaction partners); NT, no addition as no treatment; CQ, chloroquine in water.

Next, we examined whether the p62/LC3-mediated autophagy of AHR in HeLa cells can be observed in other cell types. We observed that the AHR protein levels were significantly increased to about 1.5- to 1.7-fold in the presence of 40 µM CQ for six hours in a variety of cancer cell types—namely liver cancer cell line Hep3B, lung cancer cell line A549, and two breast cancer cell lines T-47D and MDA-MB-468. These results showed that similar to HeLa cells, AHR in these cells is likely degraded via autophagy in the absence of ligand treatment (Figure 7A). Results from proximity ligation assay confirmed that AHR interacted with LC3B in the presence or absence of CQ (Figure 7B). In addition, the interaction between AHR and p62 was detected in all four cell types (Figure 7C).

### 2.6. Chaperone-Mediated Autophagy Is Unlikely Involved in the Degradation of AHR in HeLa Cells

**Figure 8:**
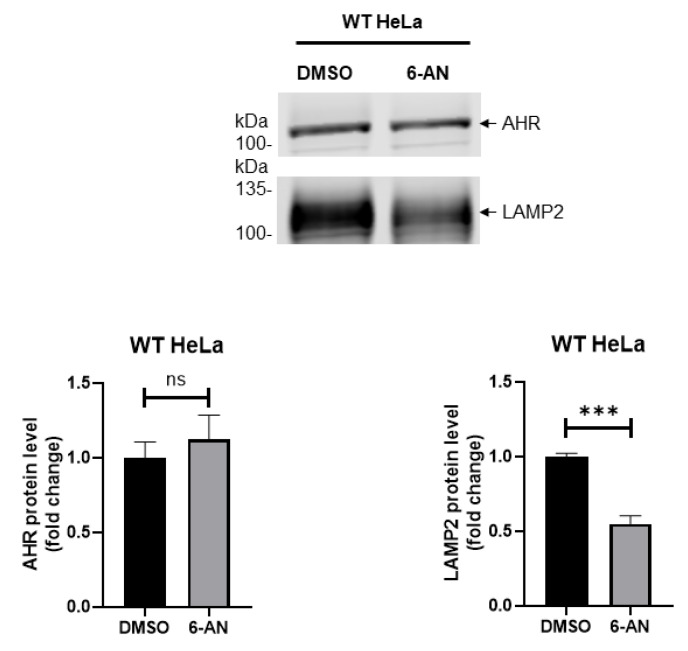
Activation of chaperone-mediated autophagy does not degrade AHR in HeLa cells. Wild type (WT) HeLa cells were treated with a chaperone-mediated autophagy activator 6-aminonicotinamide (6-AN, 100 μM) for 24 h. Treatment of 6-AN did not change AHR protein levels (bottom left graph) but reduced LAMP2 levels (bottom right graph). Images above are representative of triplicate samples in one experiment and this experiment was repeated once with similar results. Each Western lane contained 30 μg of whole-cell lysate. Data are normalized by the total protein stain. The plots represent means ± SD, *n* = 3. An unpaired *t*-test was used to determine statistical significance.

Next, we explored whether another selective autophagic pathway, namely chaperone-mediated autophagy, could degrade AHR in HeLa cells. This type of autophagy requires interaction with the lysosomal membrane-bound protein LAMP2A for the internalization of cargo protein into lysosomes for degradation. When HeLa cells were treated with 100 µM 6-AN, a chaperone-mediated autophagy activator, for 24 h, the AHR levels were not significantly altered (Figure 8). However, the LAMP2 immunoblot signal, which was detected using an antibody that recognizes both LAMP2A and LAMP2B, was significantly reduced after the 6-AN treatment. Collectively, we were not able to show any involvement of chaperone-mediated autophagy in the degradation of AHR in HeLa cells.

## 3. Discussion

We used HeLa cells for this study since HeLa cells were one of the human cell lines, we first tried that exhibit apparent p23-dependent degradation of AHR [15]. HeLa cells increase the AHR content after the treatment of an autophagy inhibitor such as CQ, Baf A1, or 3MA. CQ and Baf A1 suppress the acidification of lysosomes and the fusion of autophagosomes with lysosomes [31] whereas 3MA inhibits autophagosome formation via inhibition of class III PI3K at the early stages of autophagy [32]. The basal AHR protein can be ubiquitinated via K63-linkage and then degraded in LC3B-dependent autophagy. p62 likely acts as the cargo receptor of AHR to undergo autophagy since we can detect the AHR–p62 interaction by co-immunoprecipitation study and proximity ligation assay. Similarly, it has been reported that Dvl is degraded via the p62/LC3-mediated autophagy upon Wnt ligand activation [33]. In addition, T_H_9 cell transcription factor is selectively degraded via the p62/LC3 pathway in an Atg5-dependent manner [34]. Selectivity is largely conferred by the ubiquitination architectures of the client proteins and the preference of the cargo receptor to certain ubiquitin chains and cargoes. The chain formation including chain length, mixed, or branched chains may attach more information for the ubiquitin code. Post-translational modification of both the adaptors and the ubiquitin chains also add more layers of regulation [21,35,36]. Characterization of the ubiquitination machinery and the nature of the AHR–p62 interactions would provide a better picture of how AHR undergoes the LC3B-mediated autophagy.

As a co-chaperone of HSP90, p23 has been reported to stabilize HSP90 confirmation and assist AHR activation [37]. The expression of p23 is ubiquitous; however, there is limited, if any, information regarding the regulation of p23 gene expression. Additionally, there is no evidence showing that the p23 levels can be altered in the presence of an AHR ligand. Although p23 stabilizes AHR from degradation in cell lines [15,16], there is limited information on how p23 maintains the AHR protein levels in the absence of ligand treatment. In this study, the increase of the AHR protein levels in HeLa cells by CQ is higher when p23 is down-regulated, consistent with the notion that CQ inhibits autophagy, which is responsible for the p23-dependent AHR degradation. Proteins with a glutamine-rich region (such as AHR) are known to be more prone to aggregation, and these proteins can be degraded by autophagy [38]. Since p62/LC3 is involved in autophagic degradation of misfolded proteins [39], reduction of the p23 content in HeLa cells may allow AHR to be more prone to aggregation and become accessible for ubiquitination. This hypothesis is supported by our findings that the response of AHR to autophagy inhibition, activation, and LC3B knockdown are more pronounced in HeLa cells when p23 is down-regulated. Moreover, the interaction between AHR and p62 (or LC3B) is more apparent in p23 knockdown HeLa cells. The autophagy-related gene expression, however, did not show any significant difference between wild type and p23 knockdown HeLa cells. Although we are not aware of any report showing the correlation between p23 and autophagy activity, the basal LC3B-II levels and the autophagic flux are unambiguously higher when p23 is down-regulated in HeLa cells. Collectively, we concluded that the down-regulation of p23 promotes autophagy to selectively degrade AHR without altering the expression of any autophagy-related gene. Importantly, the down-regulation of p23 in HeLa cells causes more K63-ubiquitination of AHR in the absence of ligand treatment, revealing that reduction of the cellular p23 content to about 50% in HeLa cells is sufficient to promote the K63-ubiquitination of AHR, leading to the p62/LC3B-mediated selective autophagy.

Interestingly, it has been reported that HIF-1α—another member of the AHR PAS protein family—can be K63-ubiquitinated, and this ubiquitination leads to chaperone-mediated autophagy of HIF-1α during serum deprivation [40]. HIF-1α is constantly degraded by 26S proteasome under normoxic conditions. In the absence of serum, the KFERQ-like motif of HIF-1α interacts with HSC70; K63-ubiquitination of HIF-1α apparently causes the interaction between HIF-1α and the lysosomal membrane-bound LAMP-2A—a necessity for internalization of HIF-1α into lysosomes for degradation. We examined the possible role of chaperone-mediated autophagy in AHR protein degradation in HeLa cells. However, activation of chaperone-mediated autophagy by 6-AN did not alter the AHR content in HeLa cells. In addition, starvation of HeLa cells for four hours caused an increase of the AHR content whereas prolonged starvation in rodents is known to cause chaperone-mediated autophagy [41]. Collectively, chaperone-mediated autophagy is unlikely to be involved in the degradation of AHR in HeLa cells. Reduction of the LAMP2 levels in HeLa cells upon 6-AN treatment is surprising since activation of chaperone-mediated autophagy often increases the LAMP2A levels at the lysosomal membrane [42]. Realizing that the LAMP2 antibody we used to detect LAMP2A cannot differentiate LAMP2A from LAMP2B, we cannot rule out the possibility that LAMP2B may be suppressed by 6-AN treatment in HeLa cells.

This p62/LC3B-mediated AHR degradation is analogous to the cargo-induced model of selective autophagy, which involves the formation of the K63-ubiquitinated client proteins that are recognized by the cargo receptors [43]. Although both cargo-induced and starvation-induced autophagy involve the formation of autophagosome and fusion of the autophagosome with the lysosome, the signaling cascade and the involvement of autophagy-related (Atg) proteins are not exactly identical [21]. It is evidence that AHR can be degraded when autophagy is induced by nutrient deprivation or treatment of metformin. However, autophagy induction via nutrient deprivation quickly increases the amount of AHR. During that time, HSP90 protects the AHR protein from degradation, consistently with the literature that inhibition of HSP90 leads to client protein degradation through autophagy [44,45,46] and inhibition of HSP90 by geldanamycin results in the proteasomal degradation of AHR [47]. Down-regulation of HSP90 in HeLa cells exhibits more AHR degradation upon early nutrient deprivation; subsequently, AHR levels are steadily increased due to the up-regulation of the AHR gene transcription. We previously discovered that the protective role of p23 on the AHR levels does not require HSP90, since stable knockdown of HSP90 to 55% of its wild type content in HeLa cells does not alter the AHR levels and p23 mutants with modest HSP90 binding affinity can still effectively restore the AHR levels [23]. Although we observed that degradation of AHR occurs after 15-min treatment of HBSS in HSP90 knockdown HeLa cells, this reduction is unique to nutrient deprivation and is temporary, probably involves the general HSP90 protection of protein folding under stress; it is rather different from the previous observation that the long-term, steady-state AHR levels are not affected by the reduction of HSP90. Studying the underlying mechanisms of how HeLa cells trigger the synthesis of AHR in response to autophagy induction by nutrient deprivation might reveal a potential role of AHR in the autophagy-related diseases, such as neurodegenerative diseases and tumorigenesis.

## 4. Materials and Methods

### 4.1. Reagents

3MA, bafilomycin A1, and metformin were purchased from Cayman Chemical (Ann Arbor, MI, USA). Chloroquine, rabbit IgG, Duolink proximity ligation assay kit, and anti-LC3B rabbit IgG were purchased from Sigma (St. Louis, MO, USA). Cycloheximide, anti-AHR monoclonal mouse IgG (A-3x, used for proximity ligation assay and co-immunoprecipitation for p62 due to less interference in the region of p62 on Western analysis), anti-LAMP2 (H4B4), and anti-HSP90 goat IgG (N-17) were purchased from Santa Cruz Biotechnology (Dallas, TX, USA). HBSS was purchased from Gibco (Grand Island, NY, USA). Puromycin was purchased from Goldbio (St Louis, MO, USA). FBS was purchased from Gemini Bio (West Sacramento, CA, USA). HyClone DMEM, protein G Dynabeads, anti-p23 mouse IgG (JJ3), and all cell culture reagents (if not specified) were purchased from Thermo Fisher Scientific (Rockford, IL, USA). Direct-zol RNA kit was purchased from Zymo Research (Irvine, CA, USA). MMLV high-performance reverse transcriptase was purchased from Epicentre (Madison, WI, USA). qPCR SYBR Green supermix was purchased from Bio-Rad (Hercules, CA, USA). Zymopure maxiprep kit was purchased from Zymo Research (Irvine, CA, USA). RT^2^ Profiler PCR array plates were purchased from Qiagen (Germantown, MD, USA). K48- and K63-TUBE were purchased from LifeSensors (Malvern, PA, USA). p23 shRNA, HSP90 shRNA, and LC3B shRNA were purchased from Dharmacon (Lafayette, CO, USA). Anti-AHR rabbit IgG (SA210) and anti-p62 rabbit IgG were purchased from Enzo Life Sciences (Farmingdale, NY, USA). Anti-β-actin mouse IgG was purchased from Ambion (Austin, TX, USA). All secondary IgG and streptavidin conjugated with IRDye 800CW or 680 were purchased from LI-COR Bioscience (Lincoln, NE, USA). The pCMV-VSV-G (8454) and pCMV-dR8.2 dvpr packaging plasmid (8455) were purchased from Addgene (Watertown, MA, USA).

### 4.2. Cell Culture

All cell lines were authenticated by ATCC and were maintained at 37 °C and 5% CO_2_. HeLa, A549, and T-47D cells were cultured in DMEM supplemented with 10% fetal bovine serum, 2 mM GlutaMAX-I, 10 U/mL of penicillin, and 10 mg/mL of streptomycin. Hep3B and MDA-MB-468 cells were grown in Advanced MEM with 5% fetal bovine serum, 2 mM GlutaMAX-I, 10 U/mL of penicillin, and 10 mg/mL of streptomycin. For nutrient deprivation study, cells were rinsed with HBSS twice and then grown in HBSS for the designated period.

### 4.3. Generation of HSP90, p23, and LC3B Stable Knockdown Cells

Protocols for the generation of the shRNA-containing lentiviruses and lentiviral infection were previously described [15]. p23 knockdown HeLa cells were generated using p23-specific shRNA (#1475); HSP90 knockdown HeLa cells were generated using the HSP90α-specific shRNA (#6, 8563), and LC3B knockdown HeLa cells were generated using the MAP1LC3B-specific shRNA #153286 and #151769. In essence, AD-293 cells (about 7 × 10^5^ cells) were plated in 5 mL of medium without antibiotics in a 25 cm^2^ flask. After incubation at 37 °C and 5% CO_2_ overnight, cells reached 50%–80% confluence. Fresh medium without antibiotics was exchanged. Cells were transfected using EndoFectin reagent (2:1 DNA ratio) with the plasmids cocktail as follows: 2.5 μg of specific shRNA plasmid, 1.875 μg of the pCMV-dR8.2 dvpr packaging plasmid, and 0.625 μg of the VSV-G envelope plasmid. Fresh complete medium was exchanged 15 h later. Medium which contained the virus was collected after 24 h and stored at 4 °C. Another 5 mL of fresh complete medium was added to cells and was collected 24 h afterwards. The combined medium was centrifuged at 400 g for 5 min to remove any AD-293 cells that were inadvertently collected. The resulting supernatant was used for infection. The infection of HeLa cells was performed by first seeding cells in a 75 cm^2^ flask to 50%–70% confluence. Fresh complete medium containing 8 μg/mL of polybrene was exchanged. Supernatant containing lentiviral particles (0.5–1 mL) was then added. The fresh complete medium was exchanged 24 h after infection. The selection was started by adding 1.5 μg/mL of puromycin 48–54 h after infection. Western analysis was performed to determine the target protein levels after 2–3 passages.

### 4.4. Transient Transfection

Cells were grown in 6-well plates and transfection was initiated when cells were about 90% confluence. Cells were transfected with 2.5 μg of DNA and 5 μL of EndoFectin reagent. Fresh complete medium was exchanged 24 h after transfection. Treatment of CQ was started at 48 h post-transfection and cells were harvested at 54 h post-transfection.

### 4.5. Whole-Cell Lysate Preparation and Western Analysis

Cell pellets were resuspended using lysis buffer (25 mM HEPES, pH 7.4, 0.4 M KCl, 1 mM EDTA, 1 mM DTT, 10% glycerol, 1% NP-40, 1 mM PMSF, and 2 μg/mL of leupeptin) of 2.5× volume of cell pellets. After three cycles of freeze/thaw, lysates were kept on ice for 30 min and were then centrifuged at 16,000 g for 10 min at 4 °C. The supernatants were defined as whole-cell lysates and were subjected to BCA assay and LI-COR Western analysis. The protocol for Western analysis was described previously [48] with minor modification. Fifteen percent acrylamide gels were transferred for 3 h at 4 °C. After the wet transfer, total protein staining was performed using LI-COR Total Protein Stain. Membranes for the examination of LC3B levels for autophagic flux were dried overnight and wet with PBS before blocking. The transferred nitrocellulose membranes were blocked in PBS with 5% BSA for 1 h. Dilutions for antibodies were as follows: 1:1,000 for p23 (JJ3), p62, LC3B and AHR (A-3x); 1:2,000 for AHR (SA210); 1:5,000 for β-actin; 1:200 for HSP90 (N-17). If not specified, Western bands were normalized using total protein stain. Results were obtained and analyzed using an LI-COR Odyssey CLx imaging system.

### 4.6. RT-qPCR

RT-qPCR was performed as described previously [23]. In brief, RNA was extracted using the Direct-zol kit. Reverse transcription was performed from 0.5 to 1 μg of RNA using Epicentre MMLV reverse transcriptase. Quantitative PCR was performed with: 1 μL of cDNA from reverse transcription solution, 10 μL of Bio-Rad iTaq SYBR green supermix, and 0.8 pmol sequence-specific primers (AHR primers are OL615, 5’-ACATCACCTACGCCAGTCGC-3’ and OL616, 5’-TCTATGCCGCTTGGAAGGAT-3’ whereas β-actin primers are OL101, 5’-CCACACTGTGCCCATCTAGG-3’ and OL102, 5’-AGGATCTTCATGAGGTAGTCAGTCAG-3’) using a Bio-Rad CFX Connect real-time PCR machine with the following protocol: 40 cycles of 90 °C for 10 s/60 °C for 1 min with fluorescence readings taken at 60 °C. The 2 ^−ΔΔCq^ method [49] was used to present the normalized values. For RT^2^ Profiler PCR Array, reverse transcription was performed with 0.5 μg of RNA using Epicentre MMLV reverse transcriptase. PCR master mix (1,350 μL of Bio-Rad iTaq SYBR green supermix, 102 μL of cDNA synthesis solution, and 1,248 μL of RNase-free water) was prepared and an aliquot of 25 μL was added to each well of the RT^2^ Profiler PCR Array plate. A Bio-Rad CFX Connect real-time PCR machine was used with the following protocol: 95 °C for 10 min, followed by 40 cycles of 95 °C for 15 sec/60 °C for 1 min (ramp rate between 95 °C to 60 °C step was set as 1 °C/sec). Data analysis was performed at Qiagen GeneGlobe Data Analysis Center (www.qiagen.com/shop/genes-and-pathways/data-analysis-center-overview-page). 

### 4.7. Immunoprecipitation and Co-Immunoprecipitation Experiments

Immunoprecipitation was performed as described previously [23]. In brief, cells were lysed with the method described under 4.5. Three deubiquitylase inhibitors, namely 1,10-phenanthroline (5 mM), NEM (10 mM), and PR-619 (50 μM) were added in the lysis buffer for immunoprecipitation experiment with K48- and K63-TUBE Far-western analysis. One to two milligrams of whole-cell lysates were used for immunoprecipitation (IP) and co-immunoprecipitation (co-IP) of AHR using anti-AHR antibody SA210 (1:200 by volume) or anti-AHR antibody A-3x (1:100 by volume for AHR–p62 co-IP) for 30 min at room temperature. The pre-equilibrated Protein G Dynabeads (1:200 by volume) were then added to each sample with the assay buffer: 25 mM HEPES, pH 7.4, 0.15 M NaCl, 1 mM EDTA, 1 mM DTT, 10% glycerol, 0.1% Tween-20 for IP (0.05% Tween-20 for co-IP), and 1 mg/mL of BSA. The samples were incubated with a rotation of 60 rpm overnight at 4 °C. The beads were then washed three times for 5 min each with the assay buffer and then eluted with electrophoresis sample buffer for SDS-PAGE, followed by LI-COR Western analysis.

### 4.8. K48- and K63-TUBE and Far-Western Analysis

HeLa cells were seeded and cultured overnight in a 75 cm^2^ flask. Treat the cells with DMSO (0.1%), 3MC (1 μM), and/or MG132 (10 μM) for 2 h and then harvested with cold PBS. Cell lysates were obtained using a lysis buffer (see Section 4.5) with a 4× volume of the cell pellet. Immunoprecipitation was performed as described under 4.7, followed by Far-western analysis. K48- or K63-TUBE (1:1,000) were incubated with the nitrocellulose membrane for 1 h at room temperature and then with IRDye-800 conjugated streptavidin (1:10,000) for 2 h at room temperature. The wash step between incubation was the same as in Western analysis. Results were obtained and analyzed using an LI-COR Odyssey CLx imaging system.

### 4.9. Proximity Ligation Assay

Cells were seeded on round microscope glass coverslips placed in wells of a 12-well plate and grown to about 80% confluence. Cells were then rinsed with PBS twice and incubated with ice-cold 100% methanol for 5 min. After one rinse with PBS, cells were ready for proximity ligation assay using Duolink in situ kit. In brief, cells were incubated with Duolink blocking solution (40 μL) at 37 °C for 60 min, followed by incubation with the two antibodies (in Duolink antibody diluent) used for interaction study (40 μL) at 37 °C for 60 min using the following dilution: mouse anti-AHR A-3x (1:100), rabbit anti-p62 (1:100), and rabbit anti-LC3B (1:100). After that, we performed steps involving Duolink probe incubation, ligation, and amplification according to the manufacturer’s recommendation. Nucleus staining was performed by adding 1 μg/mL of DAPI in water to cells for a 1 min incubation. Coverslips were mounted using 6 μL of PBS and were sealed with nail polish. Samples were viewed using a KEYENCE BZ-X700 fluorescence microscope and results were analyzed by ImageJ software.

### 4.10. Statistical Analysis

GraphPad Prism 8 software (La Jolla, CA) was utilized for statistical analysis. Two-tailed unpaired *t*-test, multiple *t*-tests corrected with the Holm-Sidak method for multiple comparisons, and one-way and two-way (or mixed-model) ANOVA with Sidak, Tukey or Dunnett tests for multiple comparisons were used to determine statistical significance with *p < 0.05, **p < 0.01, ***p < 0.001, ****p < 0.0001, and ns, not significant (*p* > 0.05).

## 5. Conclusions

In summary, we uncovered a mechanism for AHR protein degradation in HeLa cells. We observed that AHR is K63-ubiquitinated and binding of AHR to p62 and LC3B-II occurs. Inhibition of autophagy increases AHR protein levels. These observations are more pronounced when p23 is down-regulated in HeLa cells, suggesting that reduction of the p23 content stimulates autophagy to degrade AHR. Other than HeLa cells, four other human cell lines also show the autophagy-dependent regulation of the AHR protein levels and exhibit AHR–LC3B-II and AHR–p62 interactions. Collectively, the AHR content is controlled by selective autophagy—a novel understanding of how human cells maintain the AHR protein levels. This mechanism can potentially be manipulated in an effort to control the AHR levels for clinical applications.

## Supplementary Materials

Supplementary materials can be found at https://www.mdpi.com/1422-0067/21/10/3449/s1.

## Abbreviations

AHR	aryl hydrocarbon receptor
CQ	chloroquine
Baf A1	bafilomycin A1
3MA	3-methyladenine
Met p23	metformin cytosolic prostaglandin E2 synthase
p23KD p62 LC3B	p23 knockdown SQSTM1 (sequestosome-1) microtubule-associated protein 1 light chain 3B
WT	wild type
CHX	cycloheximide
ActD	actinomycin D
3MC	3-methylcholanthrene
WCL	whole-cell lysate
PLA	proximity ligation assay
NC	negative control
NT	no treatment
CMA	chaperone-mediated autophagy
6-AN	6-aminonicotinamide
HBSS	Hank’s balanced salt solution
HIF-1α	hypoxia-inducible factor 1-alpha
HSC70	heat shock cognate protein of 70KDa
LAMP-2A	lysosomal associated membrane protein 2A
HSP90	heat shock protein 90
XAP2	Hepatitis B Virus-X associated protein 2
KFERQ NEM	lysine-phenylalanine-glutamate-arginine-glutamine N-ethylmaleimide
